# Amphibian community structure along elevation gradients in eastern Nepal Himalaya

**DOI:** 10.1186/s12898-019-0234-z

**Published:** 2019-05-02

**Authors:** Janak R. Khatiwada, Tian Zhao, Youhua Chen, Bin Wang, Feng Xie, David C. Cannatella, Jianping Jiang

**Affiliations:** 1 CAS Key Laboratory of Mountain Ecological Restoration and Bioresource Utilization & Ecological Restoration and Biodiversity Conservation Key Laboratory of Sichuan Province, Chengdu Institute of Biology, Chinese Academy of Sciences, Chengdu, 610041 China; 2 University of Chinese Academy of Sciences, Beijing, 100049 China; 3 Department of Integrative Biology, University of Texas, Austin, USA

**Keywords:** Amphibians, Community structure, Environmental correlation, Elevational diversity gradient, Ecology of the Himalayas

## Abstract

**Background:**

Species richness and composition pattern of amphibians along elevation gradients in eastern Nepal Himalaya are rarely investigated. This is a first ever study in the Himalayan elevation gradient, the world’s highest mountain range and are highly sensitive to the effects of recent global changes. The aim of the present study was to assess amphibian community structure along elevation gradients and identify the potential drivers that regulate community structures. Amphibian assemblages were sampled within 3 months in both 2014 and 2015 (from May to July) using nocturnal time constrained and acoustic aids visual encounter surveys. In total, 79 transects between 78 and 4200 m asl were sampled within 2 years field work. A combination of polynomial regression, generalized linear models, hierarchical partitioning and canonical correspondence analysis were used to determine the effects of elevation and environmental variables on species richness, abundance, and composition of amphibian communities.

**Results:**

Species richness and abundance declined linearly with increasing elevation, which did not support the Mid-Domain Model. Among all the environmental variables, elevation, surface area and humidity were the best predictors of species richness, abundance and composition of amphibians. The majority of amphibian species had narrow elevation ranges. There was no significant correlation between species range size and elevation gradients. However, body size significantly increased along elevation gradients, indicating that Bergmann’s rule is valid for amphibians in eastern Nepal Himalaya.

**Conclusions:**

This study indicates that eastern Nepal Himalaya is a hotspot in amphibian diversity, and it should be served as a baseline for management and conservation activities.

**Electronic supplementary material:**

The online version of this article (10.1186/s12898-019-0234-z) contains supplementary material, which is available to authorized users.

## Background

Understanding how community patterns (e.g., species richness, abundance, distribution range size, and body size variation) change along elevation gradients have been a central topic in modern ecology, biogeography, and conservation [1]. As one metric of community structure, species richness is expected to decrease with increasing elevation [2], which can be attributed to the difference of species distribution and composition. Many previous studies have demonstrated the determination of biotic and abiotic factors on species richness and distribution along elevational gradients in local communities. Specifically, climate factors (e.g., temperature and rainfall) can be considered as the first filters acting on species richness and distribution [3, 4]. Then, larger land surface area is expected to support more individuals and species under similar climatic conditions [5, 6]. Biological interactions (e.g., competition, predation, and productivity) can influence the occurrence of species and, to a greater extent, species richness [7, 8]. In amphibians habitat, the first environmental filters acting on species richness is heterogeneity [9–11]. This is because the heterogeneity of habitats can provide quantitative amphibian species different vegetation types, which associated with food resources, space, and microhabitat types [10–12]. However, habitats heterogeneity can be disturbed by human activities through pollution, degradation, and land use change (e.g., deforestation), causing the cascading effects on amphibian communities such as taxonomic homogenization and species richness decline [11–13].

Species distribution range size is another important component of community structure. It is considered to be a major factor that is highly correlated with extinction risk in organisms, and is also critical to study biotic responses to environmental factors [14]. Species with small distribution range will be more at risk as their entire range can be more easy to be affected by threatening factors [15]. More importantly, these species usually have small population, which may induce inbreeding and demographic stochasticity, and thus further enhance extinction risk in the long run [16]. Various rules have been proposed to explain the response of distribution range of species along elevation gradients [17]. Rapoport’s rule, for instance, states that species adapted to higher elevations should have a larger distribution range because of climatic tolerance [18]. Indeed, species distribution range is the fundamental unit of species richness gradients [19]. This is because species distribution range is related to elevation boundaries, and species with large distribution range must have their distribution midpoints closed to the center of the domain (i.e., elevation) [20]. As a greater number of organisms mid-range appear at the mid-elevation (e.g., plants [21]; mammals [22]; birds [3]; and fish [23], increasing overlap of species distribution range toward the centers result in the highest species richness occurred at the middle elevations [20]) (but in the convergence area, species richness increases [24]). Therefore, a hump shape relationship between species richness and elevation gradients can be detected (i.e., mid-domain effect [25]).

In addition, body size structure is a key concept with its ecological role comparing to other facets of community structures. This is because it provides information about animals life history [26], predator–prey interactions [27], and extinction risk [14] to ecologists and conservationists. To correlate the body size and environmental gradient, it is stated that organisms tend to be larger in cooler climates [28, 29] and afterward this concept has been named as Bergmann’s rule. This rule has been proved to be true for some endothermic animals (e.g., mammals and birds [3, 30]), but not always true for ectothermic animals (e.g., fish [31], reptiles [32], and amphibians [33]) which could be advantageous in cold areas to gain heat faster according to heat balance hypothesis [34]. Therefore, the test of Bergmann’s rule for amphibians in Himalayan region will help ecologists to better explain body size patterns, and to conduct amphibians conservation in this area.

Empirical studies have demonstrated the responses of different taxa along elevation gradients on mountains (see Additional file 1: Table S1). However, there is still a gap in our understanding of the elevation gradients in the amphibian community structure in eastern Nepal Himalaya. In addition, given both elevation and other environmental factors (e.g., habitat type, humidity, and canopy cover) play important roles in structuring amphibian communities [35], we argue that elevation and community data cannot be accurately dealt with using the unidimensional approach. It is better to incorporate elevation and other environmental factors to document and analyze these data, especially species richness and composition. Therefore, the objectives of the present study were to (1) explore the responses of amphibian species richness, abundance, distribution range size, and body size to elevation gradients, and to (2) quantify the environmental determinants of species richness, abundance, and composition in eastern Nepal Himalaya. Based on previous studies (e.g., Hu et al. [36] and Fu et al. [37]), we predict that amphibian species richness, abundance, and distribution range size may display hump-shape curves along the elevation gradients, while body size may increase linearly along the elevation gradients. We also predict that humidity, air temperature, canopy cover of vegetation, and land surface area could be more important to determine amphibian community structures in eastern Nepal Himalaya.

## Materials and methods

### Study area

**Fig. 1:**
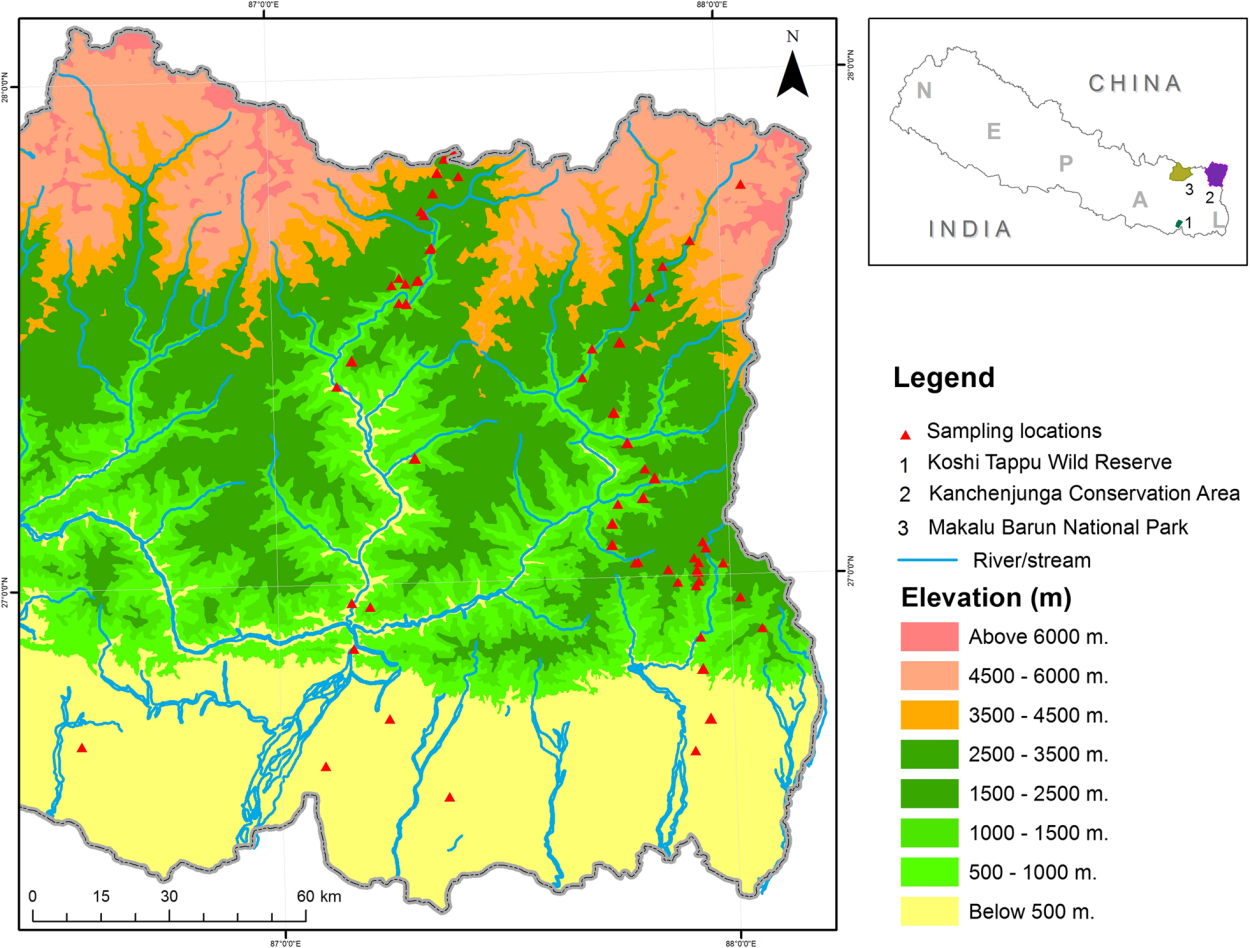
Map of the study area showing the catchment of Koshi river basin in eastern Nepal Himalayas. The triangles denote transect used for surveying amphibian

The present study was conducted in the catchment of the Koshi basin in Eastern Himalaya in Nepal (27.33805° to 26.31893°N and 86.5994° to 88.2133°E), where the elevation ranged from 78 to 3430 m above sea level (m asl; Fig. 1) within a short geographic distance (135 km). This region is characterized by rugged terrain and large climatic gradients, with the mean annual temperature is 15 °C (± 6 SD), and the annual precipitation is about 1800 mm (concentrated during monsoon season—May to October; [38]). Specifically, the study area can be divided into five distinct climatic zones [39, 40], those that correspond to specific vegetation zones. The lowland area (< 1000 m; the tropical and sub-tropical zone) is dominated by *Shorea robusta*, *Adina cordifolia*, *Dalbergia sissoo* and *Terminalia* spp. The warm temperate zone (1000 to 2000 m) is composed of evergreen broad leaf forest, which is dominated by *Schima wallichii, Castanopsis indica* and *Pinus roxburghii*. Evergreen broadleaf forest and deciduous broadleaf mixed forest dominate the cool temperate zone (2000–3000 m), with the abundant species are *Quercus* and *Rhododendron* species. In the sub-alpine zone (3000 to 4000 m), *Betula utilis* occupies the evergreen conifer forest. Above 4000 m can be considered as the alpine zone, with the vegetation dominated by *Abies spectabilis*, *Sorbus microphylla*, *Rhododendron* spp., *Salix* spp. and alpine meadows with different species of grasses [41].

### Amphibian sampling

Amphibian communities were sampled within 3 months in both 2014 and 2015 (from May to July, coincide with the rainy season) using nocturnal time constrained visual encounter and acoustic aids surveys, which is an effective method to cover entire amphibian community including terrestrial, arboreal, aquatic as well as fossorial and even well-camouflaged species [42–44]. This method involved four people systematically walking at a slow pace, intensively searching for amphibian species by turning over the stones, logs, leaf litters, tress branches, shrub and bushes along the transects (100 m × 4 m) [44]. The searches were conducted using 220 lm torches and each transect was searched for 1 h between 19:00 h and 23:00 h after the sun set every night, with one to three transects being sampled per night [44]. In total, 79 sites located in different elevations between 78 and 3430 m (Fig. 1) were sampled within 2 years field work, with each site was sampled only once in the sampling period. As amphibians are usually found near water bodies, all transects were placed nearby water sources as possible. To reduce spatial autocorrelation, all transects were separated from each other by a deep mountain gorge, stream or other prominent landmarks.

All individuals encountered were captured and stored in 15-l plastic buckets with small holes on the lid. Individuals that could not be captured were also counted. To ensure a comprehensive species list for each survey site, advertisement calls of breeding males were also recorded with a Marantz PMD670 recorder using a Sennheiser ME 66 shotgun microphone (16-bit resolution, sampling rate 44.1 kHz). Most of the species were detected by the loud and sharp calls of males [45]. All captured individuals were taken to a nearby dry place where they were photographed, identified to species and sex based on books such as Schleich and Kästle [46] and Shah and Tiwari [47], measured for body size (i.e., the maximum snout to vent length; SVL) following Olalla-Tárraga and Rodríguez [34] using a digital caliper to the nearest 0.5 mm, and released back into their original habitats. In order to prevent the transmission of diseases between individuals, new latex gloves were used for each individual during the measurement. Individuals that were difficult to identify based on morphological traits were euthanized in a chlorobutanol solution, fixed in natural formalin for 24 h and preserved in 75% ethanol. Vouchers were deposited at the Natural History Museum, Tribhuvan University, Kathmandu, Nepal. The species nomenclature herein follows that of Frost [48]. All amphibians handling and processing were in accordance with the guidelines of the Department of National Park and Wildlife Conservation, Nepal.

### Environmental variables

Environmental variables (i.e., elevation, humidity, air temperature, water temperature, canopy cover of vegetation, litter cover, land surface area, and above-ground net primary productivity) were collected based on their potential importance in shaping amphibian species composition in the field [35, 42]. These variables were obtained as follows: elevation was recorded to the nearest meter by using an altimeter (Sun Altimeter). Air and water temperature were measured at five different locations at each transect using mercury thermometer. Relative humidity was measured in percentages (five replicates within 20 m, and values were averaged for each transect) by using a digital humidity meter (Peakmeter MS6508). Canopy cover (%) of the vegetation was measured using a spherical densitometer in five locations per transect, with each location was measured toward four directions (N, S, E, W), and the averaged data was then used in statistical analyses [49]. Litter cover (%) was visually estimated in percentage at each location. Land surface area of each elevation band was calculated from a digital altitude model according to Zhang et al. [50]. Above-ground net primary productivity of each survey site can be represented as Normalized Difference Vegetation Index (NDVI), which was extracted from web page (http://earthexplorer.usgs.gov/) for 3 years (2014–2016) using ERDAS IMAGINE 9.2 (ERDAS, Norcross, GA, USA). NDVI were then averaged for the final analyses.

### Statistical analyses

Species richness was represented by the number of species, and abundance by the total number of individuals of each species. Species accumulation curves were computed using EcoSim7.0 [51] and used to test whether the sampling effort was adequate [22, 52].

We first used polynomial regressions with first order to explore the responses of species richness and abundance to elevations. The MDE null model was then computed using RangeModel 5.0 [53]. This program generates a null model for the distribution of species richness along the elevation gradients using range size and mid-points. We used 10,000 simulations without replacement to generate an interpolated richness with 95% prediction curves. The relationship between interpolated richness and elevation gradients was also explored using a linear regression. Species recorded only at one elevation point were adjusted by adding 50 m to lower elevation limit or 150 m to upper limit as described by Cardelús et al. [54] and Wu et al. [55]. Finally, Pearson correlation was used to examine the relationship between observed species richness and interpolated richness.

The range size of each species was estimated by calculating the difference between the lowest and highest elevation of its presence [22]. Polynomial regressions (first and order) were used to assess whether amphibian species’ range size follow the prediction of Rapoport’s rule [18, 56]. Linear regression was used to determine the relationship between body size (SVL) and elevations (i.e., the test for Bergmann’s rule). Only adults were included in the analyses.

Prior to multivariate analyses, variance inflation factor (VIF) was calculated to check the multicollinearity between environmental variables. High multicollinearity was detected between elevation, water temperature and air temperature [57] (Additional file 1: Table S2). Air temperature and water temperature are significantly correlated with elevation (r = − 0.947, *P* < 0.001; r = − 0.922, *P* < 0.001), both water temperature and air temperature were excluded in the multivariate regression analyses to reduce the multicollinearity [57]. Generalized linear models (GLMs) with Gaussian distribution error was used to examine the relationships between species richness/abundance and explanatory variables (elevation, humidity, canopy cover, leaf-litter cover and NDVI). The best GLM model was selected based on the lowest AIC value [58]. When required, environmental variables were log transformed prior to meet the assumptions of GLM models. These analyses were carried out using the R package MASS [59]. In addition, we also used hierarchical partitioning [60, 61] to compare the relative contribution of different environmental variables to the variation of amphibian species richness and abundance. Hierarchical partitioning (hier.part function in the R package) calculates goodness-of-fit measures according to all possible combinations of explanatory variables [62], and identifies the independent contribution of each explanatory variable [61]. The effects of environmental variables on species composition was tested using a canonical correspondence analysis (CCA) in Canoco 4.5 software [63]. A forward selection procedure with a Monte Carlo permutation test with 999 iterations was applied.

## Results

A total of 1286 individual belonging to 29 species from two orders (one Urodela and 28 Anura) and seven families were recorded in our study (Additional file 2: Table S3). The sample-based rarefaction curve attained an asymptote, indicating that the sampling effort was adequate (Additional file 2: Figure S1). Species richness per transect ranged from 0 to 12, with a mean of 3.7 ± 2.9 SD. The most abundant species were *Duttaphrynus melanostictus* (N = 193; 15.1% of all observed individuals), *Euphlyctis cyanophlyctis* (N = 182; 14.3%), *Fejervarya* sp. (N = 154; 12.1%), *Tylototriton himalayanus* (N = 118; 9.3%) and *Polypedates maculatus* (N = 104; 8.2%, (Additional file 2: Table S3). In contrast, *Microhyla taraiensis*, *Uperodon* sp., *Kaloula* sp. and *Sylvirana nigrovittata* were rare species, which occupied < 1% of the total number of captured individuals (Additional file 2: Table S3).

### Species richness and abundance along elevation gradients

**Fig. 2:**
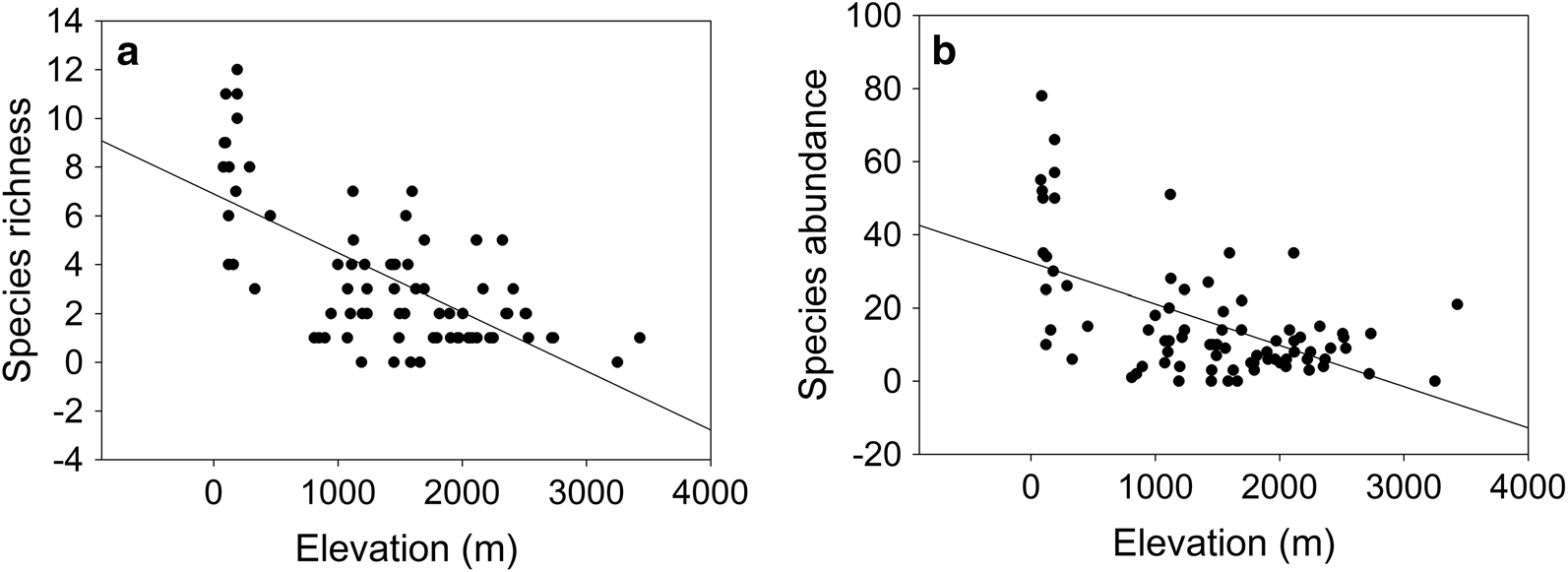
Variation of **a** species richness and **b** species abundance along the elevation gradients in eastern Nepal Himalaya

Both species richness and abundance exhibited monotonically declining trends with increasing elevations (*R*^2^ = 0.45, *P * = 0.001 and *R*^2^ = 0.31, *P * = 0.001, respectively; Fig. 2). Interpolated richness from the MDE null model showed an overall linear declining trend with elevations (*R*^2^ = 0.93, *P * = 0.001; Additional file 2: Figure S2). Moreover, observed and interpolated species richness were positively correlated with each other (*R*^2^ = 0.45, *P* = 0.001, *N * = 79).

### Species distribution range size and body size

**Fig. 3:**
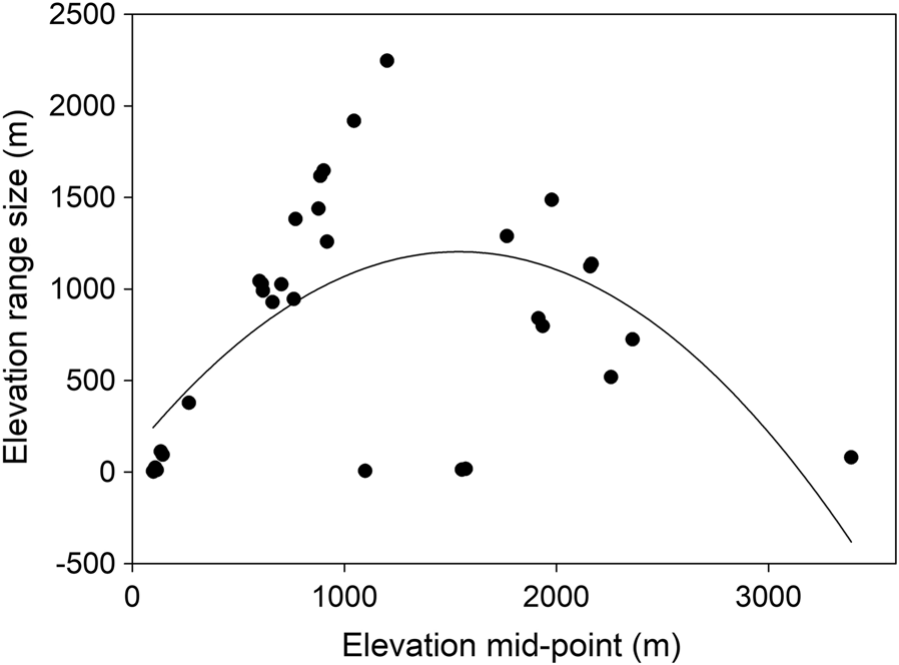
Relationship between elevation range size and elevation mid-point of each amphibian species in eastern Nepal Himalaya

About 33% of the amphibian species portrayed a narrow elevational range profile (78–500 m), 21% of the species showed wide elevational distribution (78–1800 m), and no individuals were recorded above 3450 m (Additional file 2: Figure S3). Seven species were restricted to low-elevation sites, and they were recorded only below 350 m (*Hylarana nigrovittata*, *Hoplobatrachus crassus*, *Hoplobatrachus tigerinus*, *Polypedates taeniatus*, *Uperodon globulosus*, *Spherotheca rolandae* and *Kaloula taprobanica*). Polynomial regression revealed that the species range size portrayed a curvilinear relationship (*R*^2^ = 0.38, *P *< 0.001) with elevation mid-point (average of upper and lower limit) rather than a linear one (*R*^2^ = 0.03, *P * = 0.309) (Fig. 3). This indicated that the response of amphibian species range size to elevation gradients cannot be explained by Rapoport’s elevation rule in eastern Nepal Himalaya.

**Fig. 4:**
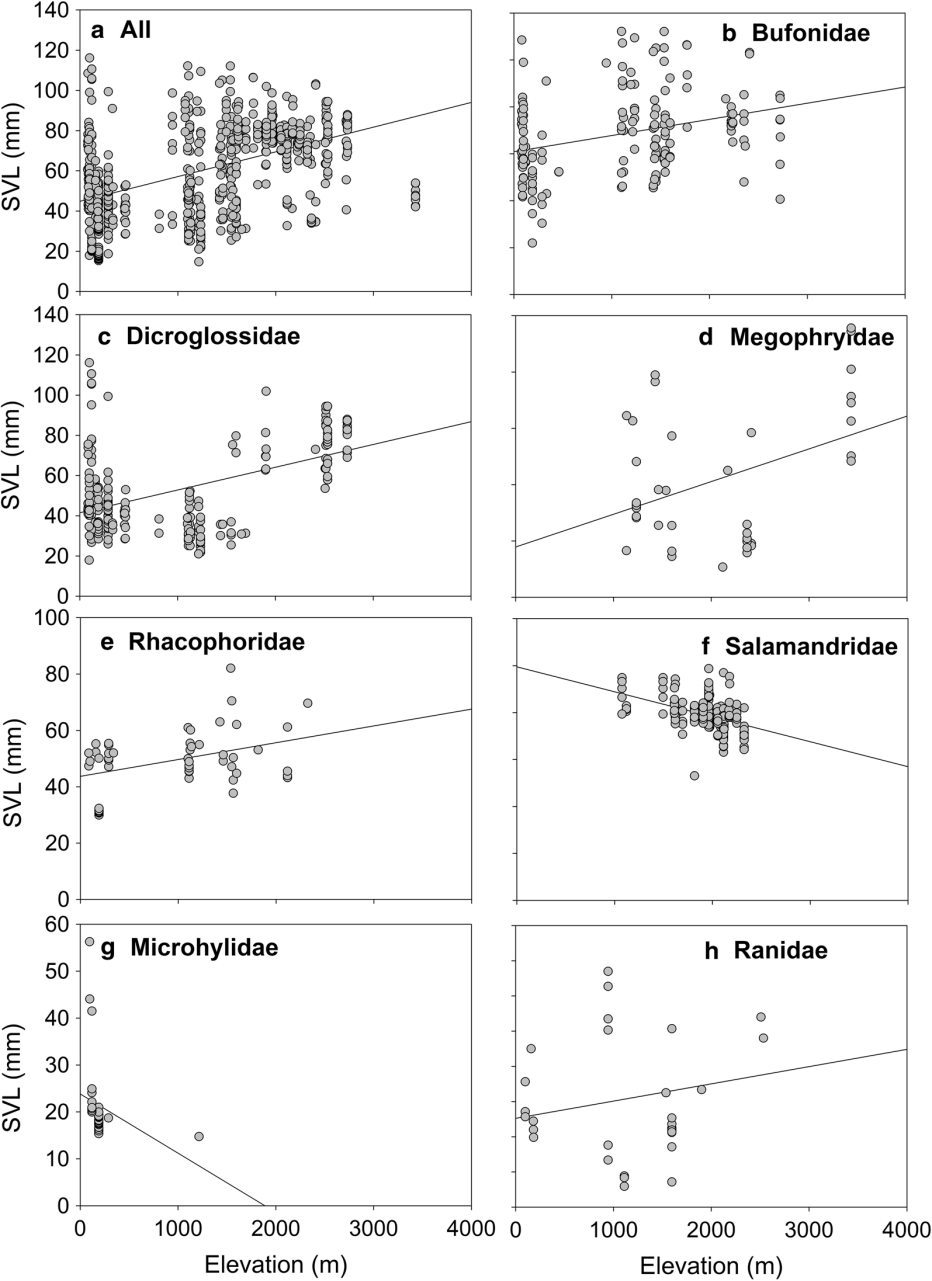
Relationships between amphibians body size and elevation in eastern Nepal Himalaya. The dots represent individuals

Amphibian body sizes have a large variation. Species with smallest body size was *Microhyla nilphamarensis* (18.28 mm ± 1.48 SD), and species with largest body size was *H. tigerinus* (83.03 mm ± 20.45 SD). Body size of all amphibians (including all the individuals) significantly increased along elevation gradients (*R*^2^ = 0.244, *P* < 0.001, Fig. 4a). Similar trend can be also detected when individuals were divided based on the sex (i.e., Male: *R*^2^ = 0.217, *P* < 0.001 and Female: *R*^2^ = 0.09, *P* < 0.001). Overall, the results suggested that amphibian species in eastern Nepal Himalaya followed Bergmann’s rule. However, at family level, only four out of seven families (Bufonidae: *R*^2^ = 0.096, *P* < 0.001; Dicroglossidae: *R*^2^ = 0.251, *P* < 0.001; Megophryidae: *R*^2^ = 0.158, *P* = 0.01; and Rhacophoridae: *R*^2^ = 0.153, *P* = 0.004, Fig. 4b–e) showed significant increasing trend whereas Salamandridae (*R*^2^ = 0.160, *P* < 0.00, Fig. 4f) showed declining trend along elevations (Fig. 6). Two families namely Ranidae (*R*^2^ = 0.045, *P* = 0.248, Fig. 4g) and Microhylidae (*R*^2^ = 0.071, *P* = 0.122, Fig. 4h) exhibited non-significant trend along elevation gradients.

### Effects of environmental variables on species richness, abundance and composition

**Fig. 5:**
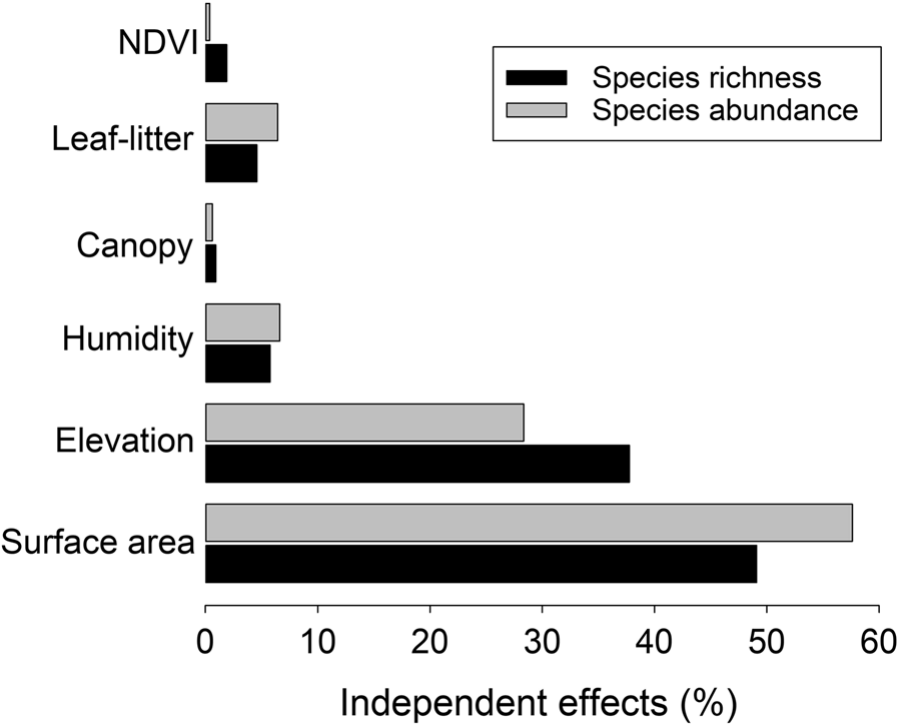
Results of hierarchical partitioning showing the independent contribution of environmental variables in the variations of amphibian species richness and abundance

Land surface area of elevation band and humidity were included in the best GLM model to explain the variation in both amphibian richness and abundance (Table 1). Specifically, both species richness and abundance had significant positive relationships with land surface area of elevation band (*P* < 0.001 and *P* = 0.001; respectively). Hierarchical partitioning analyses showed that land surface area of elevation band contributed the most to the variation of species richness and abundance (50.2% and 57.6%; respectively). While the second most important contributor was elevation, which explained 39.5% variation of species richness and 28.3% variation of species abundance, respectively (Fig. 5).

**Table 1:** Results of the generalized linear model, using species richness and abundance as the dependent variable and elevation, humidity, canopy cover, litter coverage, NDVI, area (log transformed) as the independent variables

Variable	Estimate	SE	t-value	P-value
Species richness				
Surface area (log)	7.120	0.758	9.390	0.000***
Humidity	0.034	0.024	1.427	0.158
Species abundance				
Surface area (log)	37.565	4.772	7.871	0.000***
Humidity	0.218	0.151	1.441	0.154

The asterisks denote the significance level (***P < 0.001)

**Fig. 6:**
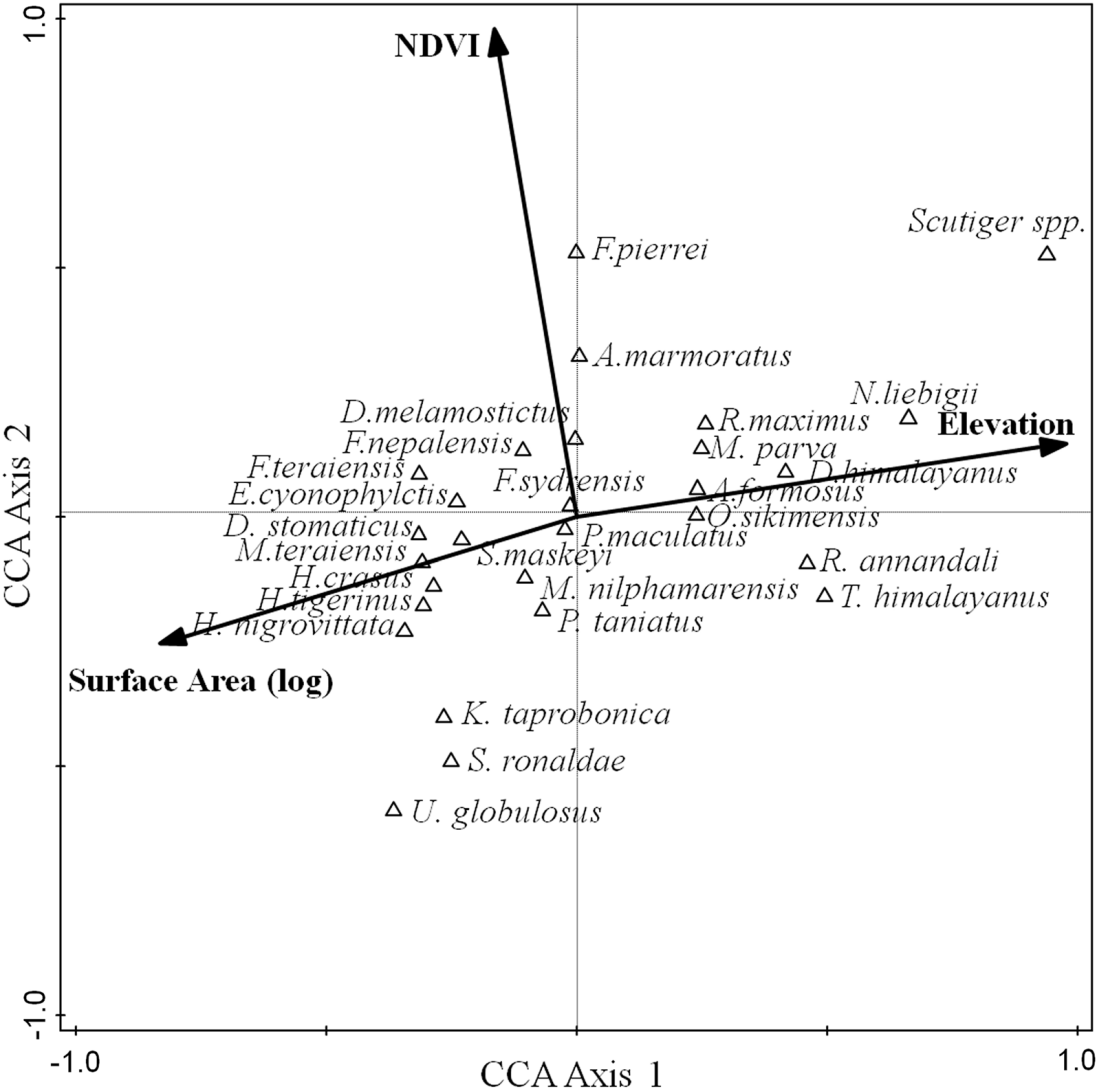
Canonical correspondence analysis (CCA) showing the relationships between environmental variables and amphibian species. Only significant variables were presented with bold lines. The length of an environmental vector indicates the degree of correlation

The CCA model revealed the significant effects of environmental factors on species composition (*P *<0.01). The first two axes explained 13.7% of the variation (10.3% and 3.4% respectively). Elevation, surface area and NDVI had a significant effect on species composition (*P *< 0.05) (Fig. 6). *T. himalayanus*, *A. formosus*, *P. annandalii*, *D. himalayanus*, *N. liebigii*, *Scutiger* spp. and *M. parva* were positively associated with elevation and negatively with land surface area of elevation band. In contrast, *K. taprobanica*, *S*. *nigrovittata*, *H. crassus*, *H. tigerinus, S. ronaldae, P. taeniatus* and *D*. *stomaicus* were positively associated with land surface areas and negatively with elevation. Some amphibian species such as *F. nepalensis*, *F. terainesis*, *F. pierrei* and *A. marmoratus* were positively influenced by NDVI (Fig. 4).

## Discussion

The present study examined amphibian community structure along elevation gradients in eastern Nepal Himalaya. Our results indicated that amphibian community structure (i.e., species richness, abundance, range size, and body size) varied significantly from low to high elevations. This is because of the different amphibian species distribution and composition which can be driven by environmental variables such as elevation, land surface area of elevation band, and NDVI [9, 50].

A total of 29 amphibian species were detected in the studied area, which covered more than 50% of the total amphibian species in Nepal [47], indicating the high amphibian species richness in eastern Nepal Himalaya. We found linear declining relationships between species richness/abundance and elevation gradients (from 12 to 0, and from 80 to 0), indicating that more amphibians prefer low elevation climate and micro-environment conditions. This is a typical pattern reported for the herpetofauna [64, 65]. In addition, previous study also confirmed the similar declining trend of reptiles richness along elevation gradients in the adjoining Eastern Sikkim Himalaya [66]. This is mainly caused by the decrease in temperature [67], with the lapse rate of temperature is estimated about 0.53 °C/100 m along elevation gradients in Nepal [68]. As the ectothermic organisms, it is widely recognized that higher temperature and precipitation in low elevation locations can usually support more species and individuals [3, 4]. And fewer amphibian species are able to survive in cold high elevation regions [69], except salamanders which will increase their species richness along elevations as many salamander species prefer cool and moist climates [70, 71]. However, the observation is in contrast with previous studies showing that amphibian species richness can exhibit a hump-shape response to elevation gradients in other mountain regions such as Hengduan Mountains, China [31] and tropical Andes [72]. This is because the mid-domain effect can be affected by sampling effort, geometric constraints on species range boundaries, and geographical scales [21, 25, 50]. In the present study, the sample-based rarefaction curve attained an asymptote, which indicated that sampling effort did not significantly influence the relationship between species richness and elevation gradients. Moreover, our results demonstrated that most of the amphibian species displayed a narrow range size instead of a uniform distribution. This is likely the main reason that amphibian species in eastern Nepal Himalaya does not follow the MDE prediction.

Moreover, the curvilinear relationship between species distribution range and elevation gradients indicated that the Rapaport’s rule also cannot used to explain elevation patterns of amphibian community structure in eastern Nepal Himalaya. This is because most species captured in the present study were elevation specialists, with their distribution range were very narrow. For example, *U. globulosus*, *Kaloula taprobonica*, *H. crassus* and *P. taeniatus* were low-elevation restricted species (< 400 m). In contrast, *Scutiger* sp. was only recorded above 3400 m. Our results supported the claims that Rapoport’s rule is a regional, but not a global phenomenon [15].

Interestingly, we found a significant correlation between body size and elevation, confirming the predictions of Bergmann’s rule for amphibians in eastern Nepal Himalaya in overall amphibian data. Indeed, Bergmann’s rule widely exists in homeothermic animals such as mammals and birds [73, 74], as these large animals have strong ability to adjust themselves to adapt to the changing environment. However, it is usually not the same case in poikilotherm animals (e.g., *Liolaemus* lizards; [32], and fresh water fish; [75]). For amphibians, empirical support for the Bergmann’s rule is still controversial [76]. Some regional amphibian species followed the Bergmann’s rule [34, 36, 77] whereas others did not [76, 78]. In the present study, we found the inverse of Bergmann’s rule for the family Salamadridae (*T. himalayanus*), which was consistent with previous findings showing the similar patterns of North-American and Europeans Urodele [34]. Therefore, future studies should focus on the mechanisms that mediate the Bergmann’s rule in amphibian species. Further, the cascading effects of Bergmann’s rule on ecosystem functioning should also be investigated.

The multivariate analyses (i.e., GLM, hierarchical partitioning and CCA) showed that land surface area of elevation band was the most important variable that affected amphibian species richness, abundance and composition in eastern Nepal Himalaya. These results complement the area-species hypothesis indicating that there are more individuals and species can be found in the elevation band with larger land surface area [5, 6, 79]). It is not surprising that humidity was the second most important variable that can influence amphibian community structure, as it is a critical factor to determine amphibian reproduction and thermoregulation [80]. More importantly, humidity also linked with water availability and is considered as the surrogate of productivity [81, 82]. And more productive habitats (NDVI) can support more species and individuals [83, 84]. This shows that the productivity hypothesis is well supported by amphibian species in eastern Nepal Himalaya [83, 84].

## Conclusions

This study indicates that eastern Nepal Himalaya is rich in amphibian diversity, which decreases along the elevation gradients. This is because lower elevation areas are larger, and they can also provide suitable habitats for amphibians (i.e., more humidity and food). Moreover, based on the curvilinear relationship between species range size and elevation gradients, our results support the claims that Rapoport’s rule is not suitable for all the taxa globally. Interestingly, our results demonstrate the acceptance of Bergmann’s rule of amphibian body size variation in eastern Nepal Himalaya. Overall, our results could provide important baseline information to design effective conservation and management strategies in the future.

## Additional files

**Additional file 1: Table S1.** Available studies on vertebrate fauna along elevation gradient in the Himalayas and its neighboring countries. **Table S2.** Summary of variance inflation factor.

**Additional file 2: Table S3.** Amphibian species with total number of individuals observed in eastern Nepal Himalaya. Numbers in parenthesis refer to total percentage contribution of each species to the total sample. **Figure S1.** Pattern of amphibian species richness observed in eastern Nepal Himalaya along elevation gradients (black filled circles), with the open circles and triangles representing the 95% upper and lower prediction values, respectively. **Figure S2.** Pattern of amphibian species richness observed in eastern Himalaya (black filled circles), with the 95% upper (open circles) and lower (open triangles) prediction curves generated from Mid-Domain null analysis in RangeModel 5.0. **Figure S3.** Estimation of elevational distribution ranges of amphibian species along elevation gradients in eastern Nepal Himalaya.
