# Supplementary material for: Amphibian community structure along elevation gradients in eastern Nepal Himalaya
Source: BMC Ecol. 2019 May 2;19:19. doi: 10.1186/s12898-019-0234-z (PMC6498630; doi:10.1186/s12898-019-0234-z)
Supplement: Supplementary file 1 — Additional file 1: Table S1. Available studies on vertebrate fauna along elevation gradient in the Himalayas and its neighboring countries. Table S2. Summary of variance inflation factor. [file 12898_2019_234_MOESM1_ESM.docx]

**Amphibian community structures along elevation gradients in Eastern Nepal Himalaya**

Janak Raj Khatiwada, Tian Zhao^*^, Youhua Chen, Feng Xie, Bin Wang, David C. Cannatella and Jianping Jiang

**Additional file 1**

**Table S1** Available studies on vertebrate fauna along elevation gradient in the Himalayas and its neighboring countries.

| Area/ region | Taxa studied | Research questions addressed | Hypotheses tested | Methods | References |
| --- | --- | --- | --- | --- | --- |
| Nepal | Birds and mammals | i) What is the altitudinal distribution pattern of birds and mammals? | - | Literature review | (1) |
| India, Sikkim Himalaya | Birds | i)How diversity of birds vary along elevation? ii) What is difference of bird community structure at highly disturbed and undisturbed forests? | - | Transects survey (100×40 m) | (2) |
| China | Fish | i)What is patterns in diversity, altitudinal range and body size of freshwater fishes along an elevational gradient in the Yangtze River basin? | Mid-domain null model, Rapoport's rule | Literature review | (3) |
| Nepal | Birds | i)What is the habitat preference and foraging ecology of alpine birds? | - | area count method | (4) |
| China , Hengduan Mountains | Frog | i)What is elevational patterns of frog species richness and endemic richness in the Hengduan Mountains of China | Mid-domain effect | Literature review | (5) |
| Ladakh, northern India | pikas and voles | i) Do pikas and voles have an impact on the spatial patterns of plant species diversity in the Trans-Himalayan rangelands? ii) Do they reduce vegetation cover? | - | Small mammal colonies with random off-colony plots | (6) |
| Hengduan Mountains, China | Lizard and snakes | i) What is the species richness patterns of lizard and snake along elevational?  ii) How of land area and climatic factors affect the distributionon lizard and snake diversity along the elevational gradient in the Hengduan Mountains? | - | Literature review | (7) |
| Western Ghats, South India | Amphibians | i)What is the patterns of anuran species richness along an elevation gradient?  What are factors that govern anuran species richness along elevational gradient? | Mid-Domain Null model | Night-time sampling using transect | (8) |
| Western Ghats, South India | Fish | i) What is the diversity and distribution patterns of fish fauna? ii) Which physical habitat features like altitude, stream reaches and microhabitats are associated for this patterns? | - | Filed sampling | (9) |
| Sikkim Himalaya, India | Reptiles | i)What is the distribution pattern of reptiles along an elevation gradient in the Eastern Himalayas and its determinants? | Mid-Domain Null model | Time constrained visual encounter survey | (10) |
| India, Sikkim Himalaya | Birds | i)What are patterns of bird diversity along elevation gradient and their determinants? | Mid-domain null model, Rapoport's rule | Open width point count method along transects | (11) |
| China | Spiny frogs | i)What are the patterns of species richness along elevational gradients? ii)What is the relationship between elevational range size of Spiny frogs and elevation? Do spiny frogs follow Bergmann’s rule? | Mid-domain null model, Bergmann’s rule, Rapoport's rule | Literature review | (12) |
| India | Fish | i)What are the patterns of fish species richness along the Himalayan elevational gradients (50–3800 m)? ii)What are the major drivers of this patterns? | - | Literature review | (13) |
| Hengduan Mountains, China | Bird | i)What are the elevational patterns in species richness for all breeding bird species? What are the roles of area, climate, productivity and geometric constraints in explaining the elevational patterns of species richness? | Mid-domain null model | Museum records and observational records | (14) |
| The Gongga Mountain, China | Non-volant small mammals | i)What determine the species richness patterns of non-volant small mammals along an altitudinal gradient, What are the potential environmental factors in shaping richness patterns along the altitudinal gradient? | Mid-domain null model | Visual observation, animal trapping | (15) |
| Nepal | Birds | i)What factors determine the species richness patterns of birds along elevation gradient? | Mid-domain null model | Literature review | (16) |
| Sikkim Himalaya, India | Butterfly | i)What are the patterns of butterfly species richness and species turn and their determinants over along the elevation gradient in Sikkim, Eastern Himalaya? | - | Point count method | (17) |
| Nepal | Amphibians | i) What is the species richness and abundance pattern of anurans? ii) What are the major factors influencing the patterns? | - | Transect method | (18) |
| Nepal | Birds | i)What are the patterns of bird species richness (functional groups) and their determinants over along the elevation gradient in central Himalayas? | - | Point count method | (19) |
|  |  | i) What are the species richness patterns of bird (functional groups)? ii) How seasonality effect on species richness patterns? | - | Point count method | (20) |
| Gyirong Valley, China | Birds | i)What are the species richness patterns of birds along elevation? ii) What are the major factors spatial and environmental factors influencing the distribution of bird species richness? | Mid-domain null model | Point count method | (21) |
| Western Himalayas | Mammals | i) What are the gamma-diversity, species richness and body mass distribution patterns of large herbivores? ii) Which biotic and abiotic factors correlate with observed richness patterns distribution of all terrestrial large-mammalian herbivores? | Mid-domain null model | Literature review | (22) |
| Nepal, India, Pakistan and China | *Scutiger* frog | i) Do the phylogenetically oldest lineages to occur in this part of the Himalaya-Tibet orogeny? | Tibetan-origin hypothesis | Molecular phylogenetic | (23) |
| Qinling Mountains, China. | Rodents | i)What are elevational distribution patterns on diversity (alpha and beta diversity) rodents and its underlying mechanisms on the southern and northern slopes? | Mid-domain null model | Visual observation, animal trapping | (24) |
| Ailao Mountains Yunnan, China | Small mammals | i) what are the mammal richness patterns along the elevational gradients of two opposite faces of the Ailao Mountains?; ii) is there any difference in between the two contrasting slopes within the same mountain range but different spatial and climatic conditions? | Mid-domain null model | Transect lines survey and animal trapping | (25) |
| Gyirong Valley, the Mount Qomolangma National Nature Reserve, China | Non‐volant small mammal | i) What is the species richness pattern of non-volant small along elevation? ii) How do pure spatial variables and spatially structured environmental variables shap the elevational richness patterns small mammal species? | Water–energy dynamics model,  MDE model | Transect lines survey and animal trapping | (26) |
| Wolong Nature, Sichuan. China | Rodents | i) Is there any relationship between species range shifts with local climate change? ii) How the selected traits of species explain the difference in distributional responses among species? | - | Animal trapping, examination of museum specimens | (27) |
| Hengduan Mountains, China | Rodents | i) Is there any relation between functional and phylogenetic signal relating to the diversity patterns of along elevation? ii) How the morphological traits vary along elevation? | Bergmann's rule and Allen's rule | Transect lines survey and secondary data | (28) |
| Gongga Mountain (Sichuan), Baima Snow Mountain (Yunnan) and Sejila Mountain (Tibet), China | Small mammals | i) What is the relationship between species mean abundance and elevational range size? ii) what is the relationship between endemic species richness and elevation? | - | Transect lines survey and animal trapping | (29) |

**Table S2** Summary of variance inflation factor

| **Environmental variables** | **Variance inflation factor (VIF)** |
| --- | --- |
| Elevation | 14.02 |
| Water temperature | 12.583 |
| Air temperature | 8.665 |
| Humidity | 1.154 |
| Canopy cover | 1.34 |
| Leaf litter cover | 1.44 |
| Distance to settlement | 1.258 |
| NDVI | 1.174 |
| Surface area (log) | 4.956 |

**References**

1. Hunter ML, Yonzon P. Altitudinal distributions of birds, mammals, people, forests, and parks in Nepal. Conservation Biology. 1993;7(2):420-3.

2. Chettri N, Sharma E, Deb D. Bird community structure along a trekking corridor of Sikkim Himalaya: a conservation perspective. Biological Conservation. 2001;102(1):1-16.

3. Fu C, Wu J, Wang X, Lei G, Chen J. Patterns of diversity, altitudinal range and body size among freshwater fishes in the Yangtze River basin, China. Global Ecology and Biogeography. 2004;13(6):543-52.

4. Laiolo P. Diversity and structure of the bird community overwintering in the Himalayan subalpine zone: is conservation compatible with tourism? Biological Conservation. 2004;115(2):251-62.

5. Fu C, Hua X, Li J, Chang Z, Pu Z, Chen J. Elevational patterns of frog species richness and endemic richness in the Hengduan Mountains, China: geometric constraints, area and climate effects. Ecography. 2006;29(6):919-27.

6. Bagchi S, Namgail T, Ritchie ME. Small mammalian herbivores as mediators of plant community dynamics in the high-altitude arid rangelands of Trans-Himalaya. Biological Conservation. 2006;127(4):438-42.

7. Fu C, Wang J, Pu Z, Zhang S, Chen H, Zhao B, et al. Elevational gradients of diversity for lizards and snakes in the Hengduan Mountains, China. Biodiversity and Conservation. 2007;16(3):707-26.

8. Naniwadekar R, Vasudevan K. Patterns in diversity of anurans along an elevational gradient in the Western Ghats, South India. Journal of Biogeography. 2007;34(5):842-53.

9. Raghavan R, Prasad G, Ali PHA, Pereira B. Fish fauna of Chalakudy River, part of Western Ghats biodiversity hotspot, Kerala, India: patterns of distribution, threats and conservation needs. Biodiversity and Conservation. 2008;17(13):3119-31.

10. Chettri B, Bhupathy S, Acharya BK. Distribution pattern of reptiles along an eastern Himalayan elevation gradient, India. Acta Oecologica. 2010;36(1):16-22.

11. Acharya BK, Sanders NJ, Vijayan L, Chettri B. Elevational gradients in bird diversity in the Eastern Himalaya: an evaluation of distribution patterns and their underlying mechanisms. PLoS One. 2011;6(12):e29097.

12. Hu J, Xie F, Li C, Jiang J. Elevational patterns of species richness, range and body size for spiny frogs. PLoS One. 2011;6(5):e19817.

13. Bhatt JP, Manish K, Pandit MK. Elevational gradients in fish diversity in the Himalaya: water discharge is the key driver of distribution patterns. PLoS One. 2012;7(9):e46237.

14. Wu Y, Colwell RK, Rahbek C, Zhang C, Quan Q, Wang C, et al. Explaining the species richness of birds along a subtropical elevational gradient in the Hengduan Mountains. Journal of biogeography. 2013;40(12):2310-23.

15. Wu Y, Yang Q, Wen Z, Xia L, Zhang Q, Zhou H. What drives the species richness patterns of non‐volant small mammals along a subtropical elevational gradient? Ecography. 2013;36(2):185-96.

16. Paudel PK, Šipoš J. Conservation status affects elevational gradient in bird diversity in the Himalaya: A new perspective. Global Ecology and Conservation. 2014;2(2014):338-48.

17. Acharya BK, Vijayan L. Butterfly diversity along the elevation gradient of Eastern Himalaya, India. Ecological Research. 2015;30(5):909-19.

18. Khatiwada JR, Haugaasen T. Anuran species richness and abundance along an elevational gradient in Chitwan, Nepal. Zoology and Ecology. 2015;25(2):110-9.

19. Basnet TB, Rokaya MB, Bhattarai BP, Münzbergová Z. Heterogeneous landscapes on steep slopes at low altitudes as hotspots of bird diversity in a Hilly Region of Nepal in the Central Himalayas. PloS one. 2016;11(3):e0150498.

20. Katuwal HB, Basnet K, Khanal B, Devkota S, Rai SK, Gajurel JP, et al. Seasonal Changes in Bird Species and Feeding Guilds along Elevational Gradients of the Central Himalayas, Nepal. PloS One. 2016;11(7):e0158362.

21. Pan X, Ding Z, Hu Y, Liang J, Wu Y, Si X, et al. Elevational pattern of bird species richness and its causes along a central Himalaya gradient, China. PeerJ. 2016;4:e2636.

22. Mishra C, Bhatnagar YV, Suryawanshi KR. Species Richness and Size Distribution of Large Herbivores in the Himalaya. The Ecology of Large Herbivores in South and Southeast Asia: Springer; 2016. p. 89-97.

23. Hofmann S, Stöck M, Zheng Y, Ficetola FG, Li J-T, Scheidt U, et al. Molecular phylogenies indicate a paleo-tibetan origin of Himalayan Lazy Toads (*Scutiger*). Scientific Reports. 2017;7(1):3308.

24. Shuai L-Y, Ren C-L, Yan W-B, Song Y-L, Zeng Z-G. Different elevational patterns of rodent species richness between the southern and northern slopes of a mountain. Scientific reports. 2017;7(1):8743.

25. Chen Z, He K, Cheng F, Khanal L, Jiang X. Patterns and underlying mechanisms of non-volant small mammal richness along two contrasting mountain slopes in southwestern China. Scientific Reports. 2017;7(1):13277.

26. Yiming H, Kun J, Zhiwen H, Zhifeng D, Jianchao L, Xinyuan P, et al. Elevational patterns of non‐volant small mammal species richness in Gyirong Valley, Central Himalaya: Evaluating multiple spatial and environmental drivers. Journal of Biogeography. 2017;44(12):2764-77.

27. Wen Z, Wu Y, Ge D, Cheng J, Chang Y, Yang Z, et al. Heterogeneous distributional responses to climate warming: evidence from rodents along a subtropical elevational gradient. BMC Ecology. 2017;17(1):17.

28. Yuanbao D, Zhixin W, Jinlong Z, Xue L, Jilong C, Deyan G, et al. The roles of environment, space, and phylogeny in determining functional dispersion of rodents (Rodentia) in the Hengduan Mountains, China. Ecology and Evolution. 2017;7(24):10941-51.

29. Zhixin W, Yongjie W, Jilong C, Tianlong C, Yuanbao D, Deyan G, et al. Abundance of small mammals correlates with their elevational range sizes and elevational distributions in the subtropics. Ecography. 2018;41(5):1–11.
