# Supplementary material for: Amphibian community structure along elevation gradients in eastern Nepal Himalaya
Source: BMC Ecol. 2019 May 2;19:19. doi: 10.1186/s12898-019-0234-z (PMC6498630; doi:10.1186/s12898-019-0234-z)
Supplement: Supplementary file 2 — Additional file 2: Table S3. Amphibian species with total number of individuals observed in eastern Nepal Himalaya. Numbers in parenthesis refer to total percentage contribution of each species to the total sample. Figure S1. Pattern of amphibian species richness observed in eastern Nepal Himalaya along elevation gradients (black filled circles), with the open circles and triangles representing the 95% upper and lower prediction values, respectively. Figure S2. Pattern of amphibian species richness observed in eastern Himalaya (black filled circles), with the 95% upper (open circles) and lower (open triangles) prediction curves generated from Mid-Domain null analysis in RangeModel 5.0. Figure S3. Estimation of elevational distribution ranges of amphibian species along elevation gradients in eastern Nepal Himalaya. [file 12898_2019_234_MOESM2_ESM.docx]

**Additional file 2**

**Table S3** Amphibian species with their number of individuals observed in eastern Nepal Himalaya. Numbers in parenthesis refer to total percentage contribution of each species to the total sample.

| **Order** | **Family** | **Scientific Name** | **Abundance (%)** |
| --- | --- | --- | --- |
| Anura | Bufonidae | *Duttaphrynus himalayanus* | 36(2.8) |
|  |  | *Duttaphrynus melanostictus* | 193(15.01) |
|  |  | *Duttaphrynus stomaticus* | 27(2.1) |
|  | Dicroglossidae | *Euphlyctis cyanophlyctis* | 182(14.15) |
|  |  | *Fejervarya* sp. | 154(11.98) |
|  |  | *Fejervarya nepalensis* | 11(0.86) |
|  |  | *Fejervarya pierrei* | 15(1.17) |
|  |  | *Fejervarya syhadrensis* | 26(2.02) |
|  |  | *Fejervarya teraiensis* | 17(1.32) |
|  |  | *Hoplobatrachus crassus* | 36(2.8) |
|  |  | *Hoplobatrachus tigerinus* | 43(3.34) |
|  |  | *Nanorana liebigii* | 56(4.35) |
|  |  | *Ombrana sikimensis* | 2(0.16) |
|  |  | *Sphaerotheca maskeyi* | 15(1.17) |
|  |  | *Spherotheca rolandae* | 6(0.47) |
|  | Megophryidae | *Megophrys parva* | 54(4.2) |
|  |  | *Scutiger* sp. | 21(1.63) |
|  | Microhylidae | *Microhyla nilphamarensis* | 51(3.97) |
|  |  | *Microhyla taraiensis* | 7(0.54) |
|  |  | *Uperodon* sp. | 3(0.23) |
|  | Ranidae | *Kaloula* sp. | 3(0.23) |
|  |  | *Amolops formosus* | 7(0.54) |
|  |  | *Amolops marmoratus* | 45(3.5) |
|  | Rhacophoridae | *Sylvirana nigrovittata* | 12(0.93) |
|  |  | *Polypedates maculatus* | 111(8.63) |
|  |  | *Polypedates taeniatus* | 13(1.01) |
|  |  | *Pseudophilautus annandalii* | 20(1.56) |
|  |  | *Rhacophorus maximus* | 2(0.16) |
| Caudata | Salamandridae | *Tylototriton himalayanus* | 118(9.18) |

**Figure S1** Sample-based species accumulation curve for amphibian species recorded in eastern Nepal Himalaya. The bars indicate 95% confidence intervals based on standard deviation.

**Figure S2** Pattern of amphibian species richness observed in eastern Nepal Himalaya along elevation gradients (black filled circles), with the open circles and triangles representing the 95% upper and lower prediction values, respectively.

**Figure S3** Estimation of elevational distribution ranges of amphibian species along elevation gradients in eastern Nepal Himalaya.
